# Paul Epstein (1943–2011): A Life of Commitment to Health and Social Justice

**DOI:** 10.1371/journal.pbio.1001284

**Published:** 2012-03-06

**Authors:** Richard Clapp

**Affiliations:** Boston University School of Public Health, Boston, Massachusetts, United States of America

## Abstract

Richard Clapp pays tribute to a pioneer in linking public health and the environment.

**Figure pbio-1001284-g001:**
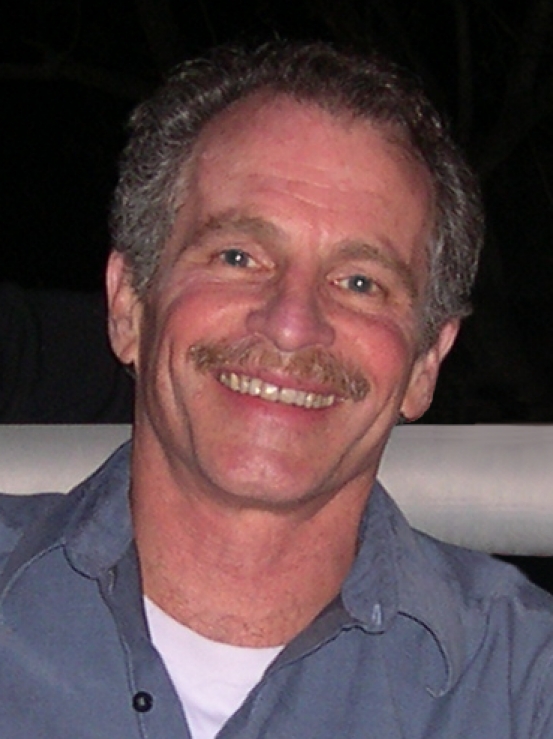
Paul Epstein. Image credit: The Center for Health and the Global Environment, Harvard Medical School.

[Fig pbio-1001284-g001]Dr. Paul Epstein, a man with a compassionate heart who had a remarkable career in public health, died at age 67 on November 13, 2011. He loved to play basketball, even though he wasn't tall, and he loved to distribute the ball to other players who had an open shot. If no one was open, he was more than willing to take the shot himself. He was a big fan of “Larry-ball,” the term some announcers used to describe the rapid passing game the Boston Celtics played during the Larry Bird era, and more recently he was a fan of the Celtics' fabulous assist leader, Rajon Rondo. The most important thing was that the points got scored. This is how Paul approached his scientific work—he was happy to assist others, but more than willing to take the lead, staking out bold positions himself if the situation called for it. The most important thing was that the work got out. And when that work revealed far-reaching links between climate change and emerging or re-emerging disease—among his most provocative, groundbreaking contributions—he went to great lengths to get the word out.

Paul Epstein grew up in New York City, in Greenwich Village. The son of a doctor and a progressive music therapist, he went to the Little Red Schoolhouse, a school committed to teaching community service whose noteworthy graduates include Robert DeNiro, Angela Davis, Dr. Tony Robbins, and Victor Navasky. Paul graduated from Stuyvesant High School and then went to Cornell University in the 1960s, where he participated in voter registration activities in Albany, Georgia, as part of the civil rights movement.

Paul was part of what a medical news magazine once called “a new breed” of physicians who were rejecting the privileges and comfortable lifestyles that seduced many of their classmates. While attending the Albert Einstein College of Medicine, Paul joined with other progressive health science students in the Bronx chapter of a national network called the Student Health Organization. He and other students organized actions and protests calling for more community-based health care, more black and Latino admissions to health science schools, and support for the community health and nutrition programs in the South Bronx. Part of this work involved support for a community-worker takeover of mental health services at Lincoln Hospital, which became a national model for bottom-up reorganization of care. The spirit of the action was, as the old saw goes, to “comfort the afflicted, while afflicting the comfortable.”

Paul and I became friends through the Student Health Organization in 1968 and we both supported the occupation of the administration offices during the student strike at Columbia University that same year. He and I, along with other medical and nursing students, set out to provide support for the strikers and alternative first aid in the event that police used excessive force while arresting those occupying the buildings. Paul had recently married Adrienne (Andy) Gates, who was working on anti-war mobilizations in New York and Washington at the time, and also participated in the Student Health Organization work.

As an intern in the innovative Kaiser health care system in the San Francisco Bay Area, Paul continued his activism and joined with colleagues who were trying to help workers organize unions in Bay Area hospitals. In 1970, after the birth of their first child, Jesse, Paul and Andy moved to a white working class community in East Boston, Massachusetts, where Paul worked as a primary care physician. He and Andy became deeply involved in campaigns to preserve community housing from airport expansion, to welcome students being bused from other neighborhoods, and to give support to a vibrant community newspaper while raising their daughter and second child, Ben.

In the late 1970s Paul and Andy, who had become a nurse, were involved in Boston-area support for the anti-colonial struggles in Southern Africa. Paul was best man at my wedding and our reception was at their house in East Boston. In lieu of wedding gifts, we asked that donations be made to the Boston Coalition for the Liberation of Southern Africa. In 1978, Paul and Andy decided to make a deeper commitment to help people in the region when they moved with their two young children to provide health care in Beira, Mozambique, under the auspices of the American Friends Service Committee.

During their two years in Beira, Paul was the director of the medical staff at the Central Hospital and Andy worked as a triage nurse. They became part of a network of international aid workers who continue to stay in touch with each other to this day. One of Paul's most harrowing experiences was watching a group of healthy young men who were members of the liberation army in nearby Rhodesia (now Zimbabwe) die before he and his colleagues could figure out the cause of their catastrophic illness. It turned out that the soldiers defending the racially segregated Ian Smith regime had secretly put rat poison in the liberation movement soldiers' uniforms, sentencing these young patients to a premature death.

Throughout the 1980s, Paul worked in a health center in largely Portuguese-speaking East Cambridge, where he practiced primary health care and was a member of the Division of Social and Community Medicine at the Cambridge Hospital. Paul continued his tradition of providing intuitive, compassionate care for his patients and serving as a role model for young physicians in training. This department had developed a reputation for some of the most innovative and effective community health care initiatives in the country. For example, the collaborative effort to prevent HIV/AIDS that Paul and colleagues Drs. David Bor and Marshall Forstein founded in Cambridge promoted creative outreach to teens and young adults most at risk.

In 1981, Paul enrolled in the Master of Public Health degree program in Tropical Public Health at Harvard School of Public Health, where he pursued his interest in the social and political determinants of disease globally. He continued to work in primary care, but also visited refugee camps in Kurdistan, under the auspices of Physicians for Human Rights, to document torture and abuse. During the same period, he and Andy worked with movements to establish accessible health care in Nicaragua and El Salvador. When a cholera epidemic began in Peru and spread throughout South and Central America in the early 1990s, Paul wrote a controversial article that linked this to the El Niño–induced warming of the Pacific Ocean and the breakdown of sanitation infrastructure. That breakdown, he wrote, was caused by the onerous requirements called structural adjustments that international lending agencies had imposed. The resulting disinvestment in public health infrastructure led to unsafe sewage management, contaminated water, and rapid spread of disease. This work is an example of Paul's broad, interdisciplinary thinking as he enlarged the scope of his inquiry into the determinants of public health.

In 1992, Paul had an experience that changed the course of his life for the next two decades. As a representative of Physicians for Social Responsibility, Paul was part of a non-governmental delegation to the United Nations Conference on Environment and Development in Rio de Janeiro, where presentations about the impacts of climate change inspired him to advocate strongly for examining their effects on health. Along with Dr. Eric Chivian, a Boston-based physician who co-founded the Nobel Prize–winning International Physicians for the Prevention of Nuclear War, Paul began a collaboration with academics Dick Levins, Howard Hu, and Mary Wilson, who studied new and re-emerging diseases around the globe. They met regularly at Harvard School of Public Health, held workshops, and wrote articles that were eventually collected in a special issue of the *Annals of the New York Academy of Sciences*. In 1994, Paul and Eric established what later became known as the Center for Health and the Global Environment (CHGE) at Harvard Medical School.

One early CHGE student project, led by Paul, examined the health impacts of oil—its extraction, refining, distribution, and combustion—around the globe. Paul and I also co-taught a course called Development and the Environment at Boston University School of Public Health. Paul gave a comprehensive overview of the interconnectedness of the global ecosystem and human health, a core theme that he continued to develop over the next decade or so. And he inspired his students to follow his example, encouraging them to do course projects out in the community, and to write final papers that challenged orthodox views and proposed bold new approaches.

In 1993, the editor of the widely read medical journal *The Lancet* asked Paul to organize a series of articles on the impact of climate change on health. This series, which ran over a period of two months, had articles by many of the leading scientists in this evolving field. Paul's own synthesis of the science of what I began to call planetary medicine was captured in an article in *Scientific American* in 2000. This article was illustrated with striking figures that showed the expanding distribution of malaria into populations at higher altitude in response to climate change, for example, and still represents some of Paul's best and most accessible recent work.

The CHGE has produced a continuing series of reports, workshops, and Congressional staff briefings in the past decade and a half. These are collected on the website (http://chge.med.harvard.edu) and constitute an outstanding legacy of Paul's and his colleagues' work. A recent report, on which I was a co-author, presents “full cost accounting for the life-cycle of coal.” This was one of Paul's most ambitious collaborative efforts and included representatives of grassroots organizations fighting mountain-top removal in Appalachia, as well as academics and advocates for coal miners' health and safety. Other reports were in collaboration with the United Nations, the reinsurance company SwissRe, NOAA, the John Merck Fund, and others, and involved scientists from Europe, Central and South America, Africa, Australia, and Asia.

I think the past few years of Paul's life were among the most satisfying for him. He was a key contributor to the Third Assessment report of the Nobel Prize–winning Intergovernmental Panel on Climate Change (IPCC). His daughter achieved notable success as a film-maker, and his son and eight-year-old grandson Izzy brought him great pleasure, in the midst of the usual ups and downs of family life. His work has had a global impact, as reflected in numerous films, television and radio interviews, media accounts, and in grateful testimony from colleagues and former students all over the world. Scientific articles he authored or co-authored have been cited thousands of times by other authors who have learned from or extended his work. He got to spend time working on his book, *Changing Planet, Changing Health*, partly in a much-loved vacation home in southern Vermont. This book (co-authored with journalist Dan Ferber) summarizes an extraordinary career and provides a much more in-depth view of eco-social impacts on health and the environment. The book concludes with Paul's prescription for moving away from a fossil-fuel economy and developing both gentler technologies and an ethic that promotes nurturance of all the species on the planet. When the book was released in April 2011, Paul got a sense of completion, even as he began chemotherapy for the non-Hodgkin lymphoma that took his life seven months later.

When the announcement of his death circulated in November, an enormous wave of condolences and emotional tributes flowed in to his family from all over the world. The list of those expressing their love and appreciation for Paul is too long to summarize adequately, and it continues to grow many months after his passing. There are plans for a memorial lecture in his name at Harvard in the fall of 2012, and additional tributes are being planned for later this year. It has been my privilege to accompany this man as his friend for over 40 years, and to get an “up close and personal” view of his career of compassion for people, patients, and the planet. His large ambitions, and his many connections and personal connectedness, are amply reflected in the recognition he has received. My only wish is that he could have been with us longer and we could have felt his joy and been inspired by his commitment even more deeply than we already have been. Paul's torch has been passed and it is up to us to keep the flame alive.

